# Molecular basis for DNA cleavage by the hypercompact Cas12j-SF05

**DOI:** 10.1038/s41421-023-00612-5

**Published:** 2023-11-21

**Authors:** Zhiqiang Duan, Xi Zhang, Jun-Tao Zhang, Shanshan Li, Ruiheng Liu, Jie Sun, Qingzhi Zhao, Nannan Jia, Ning Jia, Jian-Kang Zhu

**Affiliations:** 1 Bellagen Biotechnology Co. Ltd., Jinan, Shandong China; 2 Institute of Advanced Biotechnology and School of Life Sciences, Southern University of Science and Technology, Shenzhen, Guangdong China; 3 Department of Biochemistry, School of Medicine, Southern University of Science and Technology, Shenzhen, Guangdong China; 4 Shenzhen Key Laboratory of Cell Microenvironment, Guangdong Provincial Key Laboratory of Cell Microenvironment and Disease Research, Southern University of Science and Technology, Shenzhen, Guangdong China; 5 Key University Laboratory of Metabolism and Health of Guangdong, Southern University of Science and Technology, Shenzhen, Guangdong China; 6 Center for Advanced Bioindustry Technologies, Chinese Academy of Agricultural Sciences, Beijing, China

**Keywords:** Electron microscopy, Molecular biology

Dear Editor,

The CRISPR-Cas systems provide bacteria and archaea with adaptive immunity against foreign invading viruses and plasmids^1^. Based on their Cas gene composition, CRISPR-Cas systems are categorized into two classes with six types: Class 1 (type I, III, and IV) and Class 2 (type II, V, and VI)^1^. The compact and portable single-protein effectors from Class 2 systems, such as type II SpyCas9 (1368 aa)^2^ or type V Cas12a (~1300 aa)^3^, are widely used in genome editing, gene regulation, and genome imaging across various organisms. However, their large gene sizes pose a challenge for packaging into viral vectors, which can hinder their delivery and thus limit their utility in genome engineering applications. Cas12f family proteins represent the most compact Class 2 CRIPSR effectors (400–700 aa) reported to date and are capable of editing genes in both bacteria and human cells^3^. Distinct from Cas12f proteins that rely on the presence of both crRNA and tracrRNA and cleave target dsDNA in a dimerization-dependent manner^4,5^, the recently identified CasΦ (Cas12j) family proteins derived from huge bacteriophages rely on crRNA only and cleave target dsDNA in a monomeric form^6^. The Cas12j proteins also represent a minimal functional CRISPR-Cas system (700–800 aa), which can effectively cleave target dsDNA in vitro and mediate gene editing in human and plant cells^6–9^. Thus, Cas12j family members hold great potential for genome editing and diagnostics. While the reported Cas12j proteins exhibited efficient editing of endogenous genes in mammalian cells^10^, they displayed limited gene editing activity in plants, with gene editing efficiencies of ~0.3% and ~6% mediated by wild-type and engineered CasΦ-2 (Cas12j-2) variants, respectively, observed in the dicot plant *Arabidopsis*. In monocot plants, the reported heritable gene editing efficiency of Cas12j proteins is further limited, with only up to 1.2% in stably transformed rice lines^9^.

We aimed to identify Cas12j family members with high inherent gene editing efficiency from an environmental metagenome, to broaden the range of Cas12j-based CRISPR tools for biotechnological and agricultural applications. By searching the environmental metagenomic sequences with the conserved Ruvc nuclease domain of CasΦ2 (Cas12j-2) proteins, we identified a new hypercompact type V CRISPR-Cas system derived from *Caudoviricetes*, named Cas12j-SF05 (or CasΦ-SF05), which phylogenetically belongs to the Cas12j family (Fig. 1a). The CRISPR locus contains the Cas12j-SF05 gene encoding a 737 aa protein, and a CRISPR array that consists of 36 bp repeating sequences separated by 35–38 bp spacer sequences (Fig. 1b). The reported Cas12j family members recognize and cleave double-stranded DNA (dsDNA) by specifically recognizing a short protospacer-adjacent motif (PAM) responsible for self- vs non-self-discrimination^11^. To determine whether Cas12j-SF05 cleaves dsDNA in a PAM-dependent manner, we reconstructed the Cas12j-SF05 in complex with crRNA in vitro, and used the complex to perform a plasmid cleavage assay with a PAM library containing 6 bp randomized DNA sequences located immediately adjacent to the protospacer region, which is complementary to crRNA spacer^12^. The results showed that Cas12j-SF05^crRNA^ has a preference for a 5′-TBN-3′ PAM (B is for T, C, or G) for target dsDNA cleavage (Fig. 1c). Using fluorescently-labeled target-strand (TS) and non-target strand (NTS), we demonstrated that Cas12j-SF05^crRNA^ cleaved both TS and NTS (Fig. 1d). Sanger-sequencing of the target plasmids cleaved by Cas12j-SF05^crRNA^ revealed the generation of double-stranded breaks with staggered 5′ overhangs of 7 nt (Fig. 1d; Supplementary Fig. S1a), similar to the cleavage pattern observed in other Ca12j family memebers^3,6,8,13^. Our results showed that the Cas12j-SF05^crRNA^ cleaved dsDNA targets in a spacer length-dependent manner, exhibiting cleavage activity with a spacer length of 17 nt or more (Supplementary Fig. S1b). Furthermore, like other reported Cas12 family proteins^8^, Cas12j-SF05^crRNA^ showed non-specific trans-acting ssDNA cleavage activity upon binding of an ssDNA activator in cis (Supplementary Fig. S1c), indicating a potential for Cas12j-SF05 to be developed as a nucleic acid detecting tool for diagnostic applications.

**Fig. 1:**
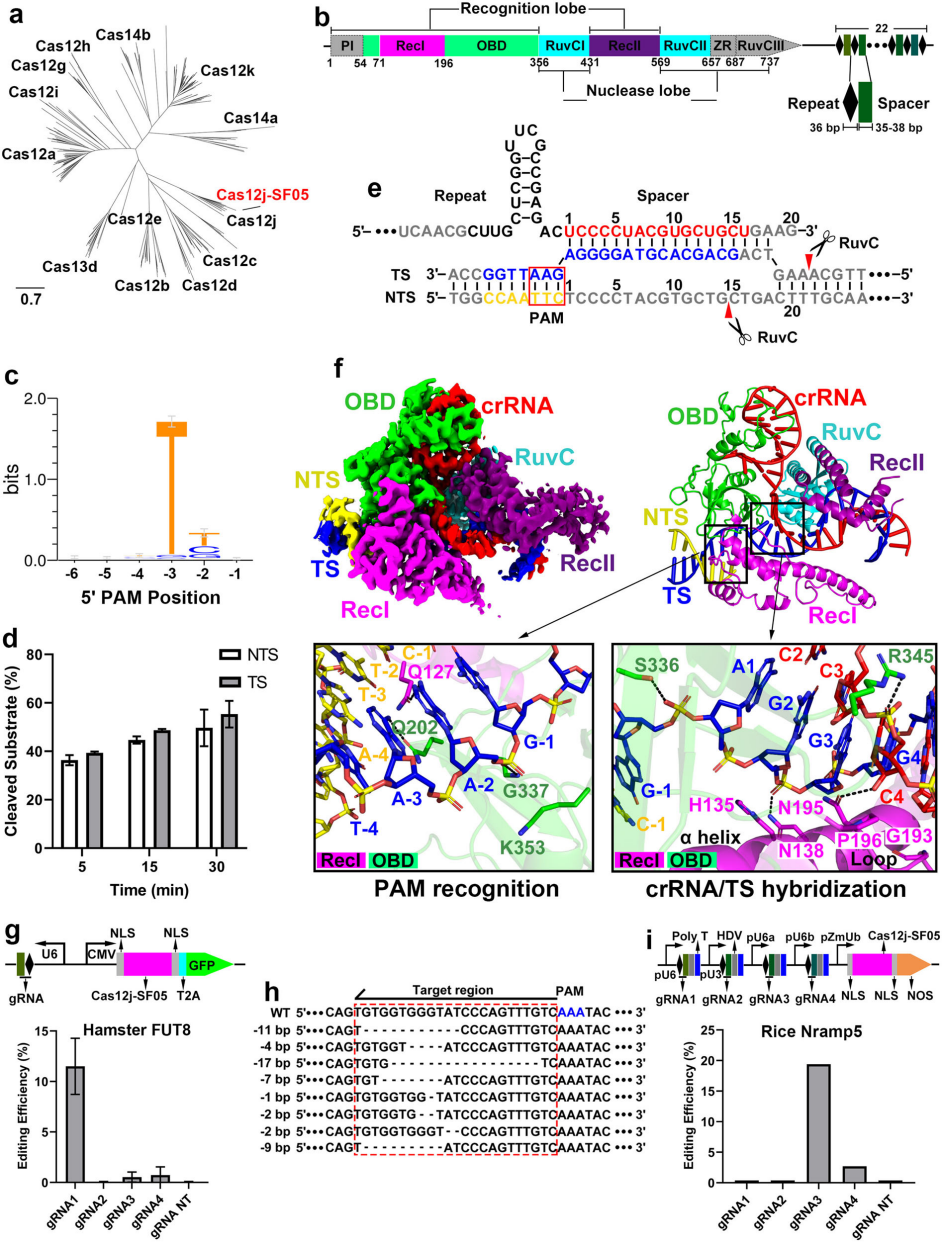
Structure-function insights into the compact type V Cas12j-SF05. **a** Maximum likelihood phylogenetic tree showing the sequence relationship between Cas12j-SF05 and other type V CRISPR-Cas12 proteins. **b** Domain organization and CRISPR array of Cas12j-SF05. The domains not observed in the Cryo-EM structure are shown in grey. **c** PAM preference of Cas12j-SF05 determined by an in vitro plasmid cleavage assay. **d** The NTS (white) and TS (grey) cleavage efficiencies of Cas12j-SF05 (*n* = 3 independent reaction replicates; data are means ± SD). **e** Schematic drawing of the sequences of crRNA, TS and NTS of target dsDNA. Segments that could be traced are in color, while disordered segments are in grey. The cleavage sites of NTS and TS are indicating by red triangles. **f** Ribbon and surface representation of the Cryo-EM structure of Cas12j-SF05^crRNA^-dsDNA ternary complex. The structural details of PAM recognition and crRNA/TS hybridization are shown in the left and right insets, respectively. **g** Target gene editing efficiency of Cas12j-SF05 at the *FUT8* gene locus in mammalian CHO cells (*n* = 3 independent reaction replicates; means ± SD). Non-targeting (NT) guide was used as a negative control. **h** The most frequent deletions for gRNA1 in the targeted region (red dashed box) within the *FUT8* gene of CHO cells. **i** Gene editing efficiency of Cas12j-SF05 at the *Nramp5* gene locus in rice plants. Non-targeting (NT) guide was used as a negative control.

To investigate the molecular mechanisms of Cas12j-SF05 ^crRNA^ recognition and cleavage of its dsDNA target, we reconstructed the dsDNA-bound Cas12j-SF05^crRNA^ complex by incubating the Cas12j-SF05^crRNA^ with a target dsDNA containing a ‘TTC’ PAM for 30 min (Fig. 1e). The complex was presumed to be in a post-cleavage state, as the target dsDNA would have been cleaved by the Cas12j-SF05^crRNA^ during the incubation period. We determined the cryo-EM structure of Cas12j-SF05^crRNA^-dsDNA ternary complex at a resolution of 3.1 Å (Fig. 1f; Supplementary Figs. S2 and S3, Table S1). The Cas12j-SF05 adopted a typical bilobed architecture, consisting of the recognition (Rec) and nuclease (Nuc) lobes, which resembles CasΦ-2 (Cas12j-2) with an RMSD of 1.534 Å over 364 Cα atoms (Supplementary Fig. S4). The significant conformational changes observed in the PAM-distal region between Cas12j-SF05 and Cas12j-2 are likely attributed to the distinct states of the complexes, as our determined Cas12j-SF05 represents a post-cleavage state, while the Cas12j-2 complex corresponds to a pre-cleavage state, in which the absence of Mg^2+^ in the Cas12j-2 complex inhibits the cleavage of the target dsDNA^6^. The Rec lobe contains several domains, including the PAM-Interacting domain (PI), the oligonucleotide binding domain (OBD), and two α-helical bundle domains (RecI and RecII). The Rec lobe is responsible for recognizing PAM sequence, stabilizing the hairpin repeat of crRNA, and forming of R-loop structure. The Nuc lobe contains a C-terminal RuvC endonuclease domain (RuvC) and an invisible zinc finger domain (ZR), which is responsible for cleaving the target dsDNA.

In our determined Cas12j-SF05^crRNA^-dsDNA ternary complex, we observed a 36-nt crRNA bound to a 22-nt traceable TS and 7-nt traceable NTS of the 55-bp dsDNA target (Fig. 1e, f; Supplementary Fig. S3). We could not observe the density of the PI domain, but we did observe that the bases of A-3 and A-2 of the TS, which are complementary to the 5′-TTC-3′ PAM, are recognized by two highly conserved glutamine residues Q202 in the OBD domain and Q127 in the RecI domain (Fig. 1f, left inset; Supplementary Fig. S5a). This specific recognition indicates the requirement of specific residues at the PAM –2 and PAM –3 positions, which is consistent with our identified ‘TBN’ PAM sequence (Fig. 1c). Mutation of residues Q127 or Q202 into alanine abolished the gene editing activity of Cas12j-SF05 in vivo (Supplementary Fig. S5b), which is consistent with the previous observations on Cas12j-2 and Cas12j-3^6,13^, supporting the critical role of these two residues in facilitating PAM recognition. Upon PAM recognition, the residue S336 in OBD domain facilitates the unwinding of target dsDNA by binding to the backbone phosphate group of G-1 (Fig. 1f, right inset). Mutation of S336 into alanine led to a ~50% reduction in gene editing efficiency in vivo (Supplementary Fig. S5b). The subsequently formed crRNA-TS duplex is further stabilized by a series of sequence-nonspecific interactions mediated by a long α helix and an adjacent loop region in RecI domain (Fig. 1f, right inset). In summary, our findings show that the miniature Cas12j-SF05^crRNA^ possesses the necessary features for target dsDNA recognition and cleavage.

To investigate the potential of Cas12j-SF05 for genome editing in eukaryotic cells, we transfected plasmids encoding several guide RNAs along with Cas12j-SF05 into CHO cells and assessed the editing efficiency towards the endogenous *FUT8* gene (Fig. 1g; Supplementary Fig. S6). Our results showed that three of the four target sites were edited, and in one instance, Cas12j-SF05 co-expressed with gRNA1 was capable of editing up to ~12% of the cells (Fig. 1g). Deep-sequencing analysis revealed that Cas12j-SF05 mainly caused 1–17 bp deletions within the target region (Fig. 1h), which is consistent with our in vitro cleavage assay results (Supplementary Fig. S1b). We next designed guide RNAs to target the endogenous *Nramp5* gene in rice and used *Agrobacterium tumefaciens* to deliver the Cas12j-SF05-gRNA plant expression plasmids into rice calli (Fig. 1i; Supplementary Fig. S6). Regenerated rice plants were sequenced to analyze the editing efficiencies in plants. We observed that two of the four target sites were edited, with one individual target showing up to 20% editing efficiency (Fig. 1i). These findings demonstrated that Cas12j-SF05 is capable of editing endogenous genes in mammalian cells and efficiently generating mutations in monocot plants, highlighting its great potential for genome editing applications.

In summary, we identified an efficient CRISPR-Cas system derived from *Caudoviricetes*, Cas12j-SF05, which belongs to the hypercompact typeV Cas12j subfamily. Our comprehensive in vitro and in vivo studies, together with structural data, shed light on the dsDNA targeting mechanism of this miniature Cas12j-SF05 CRISPR system. Our results showed that Cas12j-SF05 is capable of efficiently editing endogenous genes in both mammalian and plant cells. Notably, Cas12j-SF05 possesses inherent high editing efficiencies in monocot plants, indicating its great potential as a valuable gene editing tool in agricultural biotechnology. Our findings not only contribute to understanding the dsDNA-targeting mechanisms of this miniature Cas12j-SF05^crRNA^ complex, but also reveal the potential for developing Cas12j-SF05 as a useful gene editing tool for biotechnological and agricultural applications.

### Supplementary information

Supplementary_Information

Supplementary Table S2

## Data Availability

The cryo-EM density map (EMD-35595) and corresponding atomic coordinate (8INB) of the Cas12j-SF05^crRNA^-dsDNA ternary complex have been deposited in the Electron Microscopy Data Bank (EMDB) and Protein Data Bank (PDB), respectively.
