# Supplementary material for: Molecular basis for DNA cleavage by the hypercompact Cas12j-SF05
Source: Cell Discov. 2023 Nov 21;9:117. doi: 10.1038/s41421-023-00612-5 (PMC10663539; doi:10.1038/s41421-023-00612-5)
Supplement: Supplementary file 1 — Supplementary_Information [file 41421_2023_612_MOESM1_ESM.pdf]

Supplementary information

Materials and Methods

Cas12j detection and phylogenic analysis

Metagenomic sequences were downloaded from DOE JGI IMG/M database[1]. Firstly, CRT[2] and Prodigal[3] were used to identify the CRISPR arrays and predict the proteins, respectively. Then, Cas12j proteins[4] were used to identify Cas12j-containing candidates from downloaded Metagenomic sequences by using BLASTP program. New identified CRISPR-Cas12j systems were checked manually. For phylogentic analysis, sequences of reported class 2 type V CRISPR-Cas nucleases were collected from UniProt database by searching the keyworks of individual nuclease, respectively. These protein sequences are listed in Supplementary Table S2. Sequence alignment of Cas12j-SF05 with other Cas protein sequences was generated using MAFFT[5]. The phylogenetic tree was inferred using IQ-TREE2[6].

Plasmids construction

The protein sequence was in the supplementary file. And pET-28a overexpression vector was used for the recombinant protein expression. In brief, the full-length ORFs of Cas12j-SF05 was synthesized from TSINGKE Biological Technology and cloned into the expression vector pET-28a, which contained an N-terminal 6 × His tag. For *in vitro* plasmid cleavage, the pUC19-based plasmid containing target sequence was constructed via homologous recombination. This plasmid was used to determinate the cleavage site and spacer length of Cas12j-SF05. For cell editing assay, the Human codon-optimized Cas12j-SF05 gene synthesized from TSINGKE Biological Technology and cloned to pcDNA3.3 plasmid to generate pCAG_NLS-Cas12j-SF05-NLS-T2A-GFP_pA_pU6_BbsI by Gibson Assembly. The spacer sequences were annealed and ligated to BbsI site. Cas12j-SF05 plant expression vectors were constructed as previously described[7]. The plasmid sequences are listed in Supplementary Table S2.

Protein expression and purification

Cas12j-SF05 expression plasmid was transformed into *E. coli* BL21 (DE3), a single colony was picked to inoculate a 200 mL LB culture at 200 RPM and 37 °C. This culture was further used to inoculate 12×1000 mL LB medium which was incubated at 200 RPM and 37 °C until OD600 of ~ 0.6 was reached. The Cas12j-SF05 proteins expression was induced by adding isopropyl-β-D-1-thiogalactopyranoside to the concentration of 0.5 mM at 16 °C for 16 hrs. Cells were harvested by centrifugation at 5,000 ×g for 10 min and resuspended in lysis buffer (25 mM Tris-HCl pH 8.0, 500 mM NaCl, 20 mM imidazole, 5% glycerol). The harvested cells were lysed by sonication and centrifuged for 60 min at 20,000 rpm. The supernatant was loaded onto a 5-ml Ni-

beads (Smart-lifesciences) and washed with 20 column volumes (CV) wash buffer (25 mM Tris-HCl pH 8.0, 500 mM NaCl, 50~80 mM imidazole, 5% glycerol) and finally eluted with 4 CV elution buffer (25 mM Tris-HCl pH 8.0, 500 mM NaCl, 500 mM imidazole, 5% glycerol). The eluted proteins were concentrated to about 1 mL and further purified over a Superdex 200 10/300 GL column (GE Healthcare). Peak fractions were assessed by gel electrophoresis, and were pooled and concentrated, flash frozen in liquid nitrogen and stored at -80 °C.

In vitro assembly of Cas12j-SF05^{crRNA}-dsDNA ternary complex

To assemble the Cas12j-SF05^{crRNA}-dsDNA ternary complex, the purified Cas12j-SF05 proteins without nucleic acids were diluted to 2.0 mg/mL and mixed with crRNA (5'-GCCGUCAACGUUCAACGCUUGCUCGGUUCGCCGAGACUCCCCUACGUGCUGCUGAAG -3') at a molar ration of 1:2. After incubated on ice for 30 min, pre-annealed dsDNA target (TS: 5'-CTGCCCTTGCAAAGTCAGCAGCACGTAGGGGAGAATTGGCCA -3', NTS: 5'-TGGCCAATTCTCCCCTACGTGCTGCTGACTTTGCAAGGGCAG -3') was added at a molar ration of 1:2. The sample was then loaded on a Superdex 6 Increase 10/300 GL column (GE Healthcare) which was pre-equilibrated in PBS buffer adding with 2 mM DTT. Peak fractions were assessed by gel electrophoresis, and were pooled and concentrated, flash frozen in liquid nitrogen and stored at -80 °C.

Cryo-EM sample preparation and data acquisition

A 3 μL concentrated Cas12j-SF05^{crRNA}-dsDNA sample was loaded onto a glow discharged 300 mesh R1.2/1.3 gold holey carbon grids (Quantifoil). After a wait time of 3.0 s, grids were blotted for 3.0 s under 100% humidity at 4 °C before being plunged into liquid ethane by Vitrobot (FEI). Micrographs were acquired on a Titan Krios microscope (FEI) operated at 300 kV with a K3 summit direct electron detector (Gatan), yielding a pixel size of 0.66 Å. The defocus range was set from −1.0 to −2.5 μm. Each micrograph was dose-fractionated to 32 frames under a dose rate of 20 e^-/pixel/s, with a total exposure time of 1.08 s. A total of 5806 movies were collected.

Cryo-EM data processing

Image processing were performed by cryoSPARC v3.1[8]. A total of 523,828 particles were auto-picked using Laplacian-of-Gaussian and extracted using cryoSPARC v3.1. After several iterations of 2D classifications, particles from the good classes were selected and subjected to 3D reconstruction with the initial model generated by cryoSPARC v3.1 as a reference. Particles corresponding to the best class (70,941) were selected and subjected to non-uniform refinement in cryoSPARC v3.1. All the cryo-EM reconstructions were estimated with the Gold standard Fourier shell correlation using

the 0.143 threshold[9]. Local resolution estimates were calculated from two half data maps in cryoSPARC v3.1. The details related to data processing were shown in Supplementary Fig. 2.

Model building and refinement

With the assistance of bulky residues and PSIPRED secondary structure prediction[10] together with structural models predicted by Robetta (https://robetta.bakerlab.org/), we manually built the atomic models interactively in COOT[11]. The real-space refinement in PHENIX[12] was used to refine all models against the cryo-EM maps by applying geometric and secondary structure restraints. All structure figures were prepared in PyMol (http://www.pymol.org) and ChimeraX[13].

Identification of PAM preferences

The plasmid library of 6 bp randomized nucleotides upstream of the 5' end of the target sequence was constructed as previously described[14]. One microgram of plasmid DNA library with 6 bp randomized PAM was cleaved with 100 nM Cas12j-SF05 proteins and 250 nM crRNA in a cleavage buffer (Tris-HAC 40 mM, $Mg(HAC)_2$ 30 mM, BSA 120 μg/ml, DTT 12 mM, pH 7.0) for 60 min at 37 °C in a 100 μL reaction volume. Then, the staggered ends of the cleaved plasmid library were repaired by adding T4 DNA polymerase (Thermo Fisher Scientific) and 1 μL of 10 mM dNTP mix (Thermo Fisher Scientific) for an additional 20 min at 72 °C. These reaction products were purified with a FastPure Gel DNA Extraction Mini Kit (Vazyme Biotech, Nanjing, China), then the purified end repaired cleaved products (100 ng) were ligated with dsDNA adapters (100 ng) containing 3' dT overhang for 1 h at 22 °C using T4 DNA ligase (Thermo Fisher Scientific). After ligation, PCR amplification was performed to enrich the cleaved products containing the PAM sequence for Illumina deep sequencing. After sequencing, the reads containing 10 nt 5' sequence of the 6 bp PAM region and adapter were extracted. The frequency of each PAM was calculated and normalized to the total reads for each sample. Enriched PAMs which were considered significant > 2.5-fold from the negative control (experiments setup without functional Cas12j-SF05 complex) were used to produce PAM sequence logos by WebLogo[15]. The details related to identify of PAM preferences were shown in Supplementary Data S1.

***In vitro* cleavage assay**

For cleavage assays, the labeled dsDNA substrate was prepared by PCR extension using the target plasmids as the templates and 5'-FAM-labeled forward primers, 5'-ROX-labeled reverse primers. Then the extended products were purified by FastPure Gel DNA Extraction Mini Kit (Vazyme Biotech, Nanjing, China). Cas12j-SF05

ribonucleoprotein complexes were assembled by mixing 50 nM Cas12j-SF05 protein with 100 nM crRNA and then incubated with 300 ng labeled dsDNA in cleavage buffer (Tris-HAC 40 mM, $Mg(HAC)_2$ 30 mM, BSA 120 μg/ml, DTT 12 mM, pH 7.0) in a total volume of 60 μL at 37 °C and aliquots were collected at the following time points: 5 min, 15 min, 30 min. The cleavage products were analysis on ABI 3730xl DNA analyzer (Applied Biosystems). Fragment sizes were determined using the fragment analysis program Peak Scanner Software v1.0 (Applied Biosystems).

For *trans*-cleavage assay, 50 nM Cas12j-SF05 RNP was first incubated with 50 nM crRNA and 250 nM ssDNA activator. Then, 500 nM ssDNA-FQ (FAM-ssDNA-BHQ1) reporter of different length was mixed into the reaction buffer (40 mM Tris-HAC, 30 mM $Mg(HAC)_2$, 120 μg/ml BSA, 12 mM DTT, pH 7.0) in a total volume of 20 μL. The reaction was incubated at 37 °C for 120 min in the QuantStudio™ 3 Flex Real-Time PCR System (Thermo Fisher Scientific). Fluorescence was detected every minute (λ^{ex}: 485 nm; λ^{em}: 535 nm), and reactions without target DNA were used to establish the background.

Determination of cleavage sites

To determine the cleavage sites of Cas12j-SF05, 50 μL cleavage reaction containing 100 nM Cas12j-SF05 protein, 250 nM crRNA and 500 ng plasmid containing the target sequence were mixed in the cleavage buffer (40 mM Tris-HAC, 30 mM $Mg(HAC)_2$, 120 μg/ml BSA, 12 mM DTT, pH 7.0) at 37 °C for 60 min and quenched at 85 °C for 10 min. Then, the cleavage plasmids were purified by FastPure Gel DNA Extraction Mini Kit (Vazyme Biotech, Nanjing, China) and determined the sequences by the Sanger method.

Genome editing in CHO cells

For *FUT8* gene activation editing assay in CHO cells, the Chinese hamster ovary cells (cell line CHO-K1 (CCL-61, ATCC)) were cultured in DMEM (Gibco) supplemented with 10% (v/v) FBS (Gemini) and 1% (v/v) penicillin streptomycin (Gibco) in an atmosphere of 5.0 % CO_2 at 37 °C. About 1.0×10^5 cells were seeded onto the each well of 24-well plate and cultured until confluency reached about 70%. 600 ng pCAG-T2A-EGFP plasmid expressing Cas12j-SF05 with guide RNAs which targeted the endogenous *FUT8* gene was transfected into the cells using Lipofectamine 3000 (Invitrogen) according to the manufacturer's recommended protocol. Two days after transfection, cells were digested by Trypsin-EDTA (0.05%) (Gibco) and were centrifuged at 135 g for 3 min. 80,000 GFP-positive CHO cells were sorted via BD FACSMelody™ Cell Sorter for subsequent analysis.

To determine the length of spacer required by Cas12j-SF05 for target dsDNA cleavage *in vivo* , we inserted FUT8-gRNA1 target site into the GFP reporter system[16]. Plasmids with dirffering spacer lengths were constructed and respectively co-transformed into the CHO cells along with the GFP reporter plasmid. The fluorescence intensities of GFP, CFP and tdTomato were quantified using BD FACSMelody™ flow-cytometer for subsequent analysis.

Targeted deep sequencing

The genomic DNA of GFP-positive CHO cells was isolated using the Quick Extract DNA Extraction Solution (QE09050, Epicentre). Targeted sequence primers were synthesized and used in nested PCR amplification by Phanta Max Super-Fidelity DNA Polymerase (Vazyme Biotech, Nanjing, China). The resulting PCR products (220~230 bp) were sequenced by next-generation sequencing (NGS) and analyzed with the Hi-TOM platform[17]. The filter threshold of Hi-TOM assay was set as 15%.

Genome editing in rice

Guides were designed to target the *Nramp5* gene in rice, The Xiushui 134 calli were used for plant transformation by *Agrobacterium* strain EHA105 (transformation of rice mediated by *Agrobacterium tumefaciens*)[18]. After 4 weeks of rooting, the genomic DNA of regenerated rice plants were extracted and the target sequences were amplificated by PCR. The resulting PCR products (220~230 bp) were sequenced by next-generation sequencing (NGS) and analyzed with the Hi-TOM platform[17]. The filter threshold of Hi-TOM assay was set as 15%.

Supplementary references

1 Markowitz VM, Chen IMA, Chu K et al. IMG/M: the integrated metagenome data management and comparative analysis system. Nucleic Acids Research 2012; 40:D123-D129.

2 Bland C, Ramsey TL, Sabree F et al. CRISPR Recognition Tool (CRT): a tool for automatic detection of clustered regularly interspaced palindromic repeats. Bmc Bioinformatics 2007; 8.

3 Hyatt D, Chen GL, LoCascio PF, Land ML, Larimer FW, Hauser LJ. Prodigal: prokaryotic gene recognition and translation initiation site identification. Bmc Bioinformatics 2010; 11.

4 Pausch P, Al-Shayeb B, Bisom-Rapp E et al. CRISPR-CasΦ from huge phages is a hypercompact genome editor. Science 2020; 369:333-337.

5 Katoh K, Standley DM. MAFFT Multiple Sequence Alignment Software Version 7: Improvements in Performance and Usability. Mol Biol Evol 2013; 30:772-780.

6 Nguyen LT, Schmidt HA, von Haeseler A, Minh BQ. IQ-TREE: A Fast and Effective Stochastic Algorithm for Estimating Maximum-Likelihood Phylogenies. Mol Biol Evol 2015; 32:268-274.

7 Ma XL, Zhang QY, Zhu QL et al. A Robust CRISPR/Cas9 System for Convenient, High-Efficiency Multiplex Genome Editing in Monocot and Dicot Plants. Mol Plant 2015; 8:1274-1284.

8 Punjani A, Rubinstein JL, Fleet DJ, Brubaker MA. cryoSPARC: algorithms for rapid unsupervised cryo-EM structure determination. Nat Methods 2017; 14:290-296.

9 Rosenthal PB, Henderson R. Optimal determination of particle orientation, absolute hand, and contrast loss in single-particle electron cryomicroscopy. Journal of Molecular Biology 2003; 333:721-745.

10 Buchan DWA, Minneci F, Nugent TCO, Bryson K, Jones DT. Scalable web services for the PSIPRED Protein Analysis Workbench. Nucleic Acids Research 2013; 41:W349-W357.

11 Emsley P, Cowtan K. Coot: model-building tools for molecular graphics. Acta Crystallogr D 2004; 60:2126-2132.

12 Adams PD, Afonine PV, Bunkoczi G et al. PHENIX: a comprehensive Python-based system for macromolecular structure solution. Acta Crystallogr D Biol Crystallogr 2010; 66:213-221.

13 Pettersen EF, Goddard TD, Huang CRC et al. UCSF ChimeraX: Structure visualization for researchers, educators, and developers. Protein Science 2021; 30:70-82.

14 Karvelis T, Gasiunas G, Young J et al. Rapid characterization of CRISPR-Cas9

protospacer adjacent motif sequence elements. Genome Biology 2015; 16.

15 Crooks GE, Hon G, Chandonia JM, Brenner SE. WebLogo: A sequence logo generator. Genome Res 2004; 14:1188-1190.

16 Yang, Y., Liu, S., Cheng, Y. et al. Highly Efficient and Rapid Detection of the Cleavage Activity of Cas9/gRNA via a Fluorescent Reporter. Appl Biochem Biotechnol 2016; 180, 655–667.

17 Liu Q, Wang C, Jiao XZ et al. Hi-TOM: a platform for high-throughput tracking of mutations induced by CRISPR/Cas systems. Sci China Life Sci 2019; 62:1-7.

18 Hiei Y, Ohta S, Komari T, Kumashiro T. Efficient Transformation of Rice (Oryza-Sativa L) Mediated by Agrobacterium and Sequence-Analysis of the Boundaries of the T-DNA. Plant J 1994; 6:271-282.

Supplementary Fig. S1

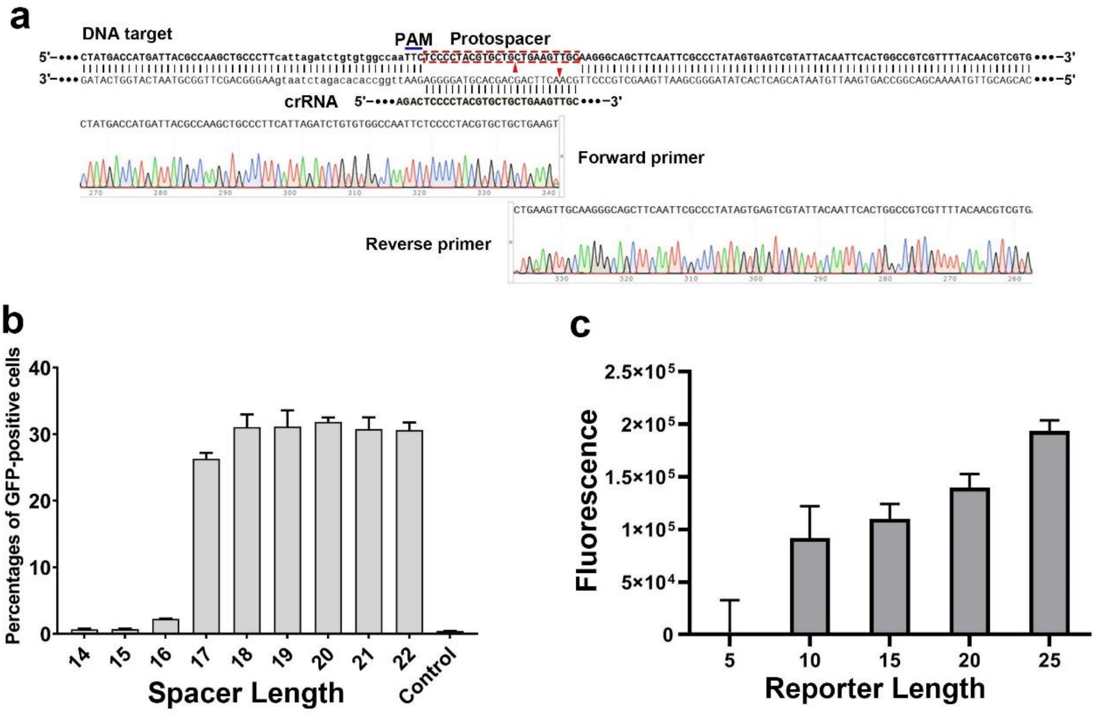

Fig. S1 Cas12j-SF05 cleaves target dsDNA *in vitro* to produce staggered cut ends and cleaves ssDNA *in trans* when activated *in cis*.

a. The identification of the Cas12j-SF05 cleavage sites. Sanger-sequencing traces are shown below. The cleavage sites are indicated by red triangles.

b. *In vivo* target dsDNA cleavage assay using Cas12j-SF05 with crRNAs containing different spacer lengths (n = 3 independent reaction replicates; mean ± s.d.).

c. Cas12j-SF05 cleaved ssDNA of different lengths *in trans* when activated by ssDNA activator *in cis.* (n = 3 independent reaction replicates; mean ± s.d.).

Supplementary Fig. S2

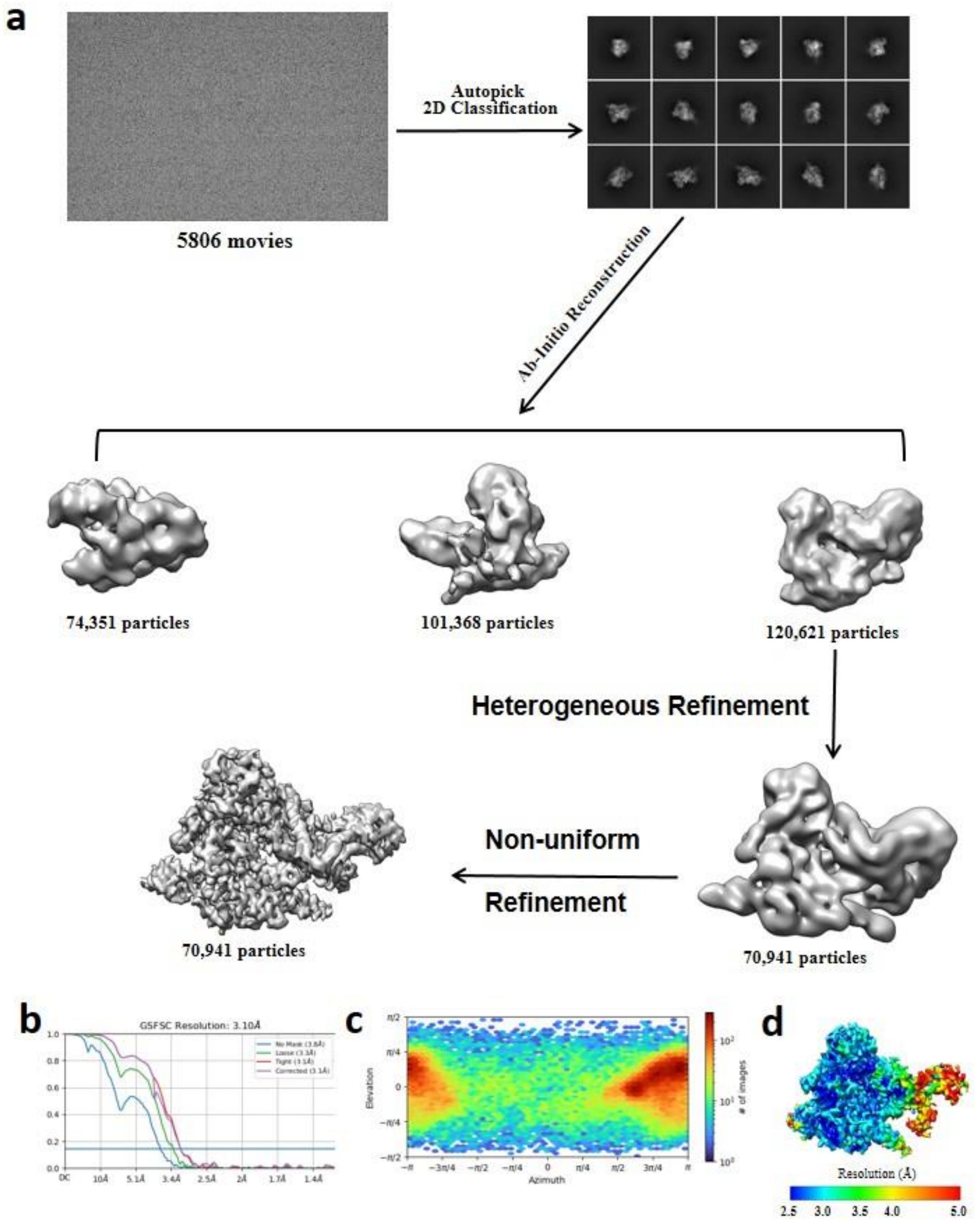

Fig. S2 CryoEM reconstruction of Cas12j-SF05^{crRNA}-dsDNA ternary complex.

a. Flow chart of image processing for Cas12j-SF05^{crRNA}-dsDNA ternary complex.

b. Fourier Shell Correlation curve of the Cas12j-SF05^{crRNA}-dsDNA ternary complex.

c. Direction distribution plot of the Cas12j-SF05^{crRNA}-dsDNA ternary complex.

d. Final three-dimensional reconstructed map of the Cas12j-SF05^{crRNA}-dsDNA ternary complex, colored according to local resolution.

Supplementary Fig. S3

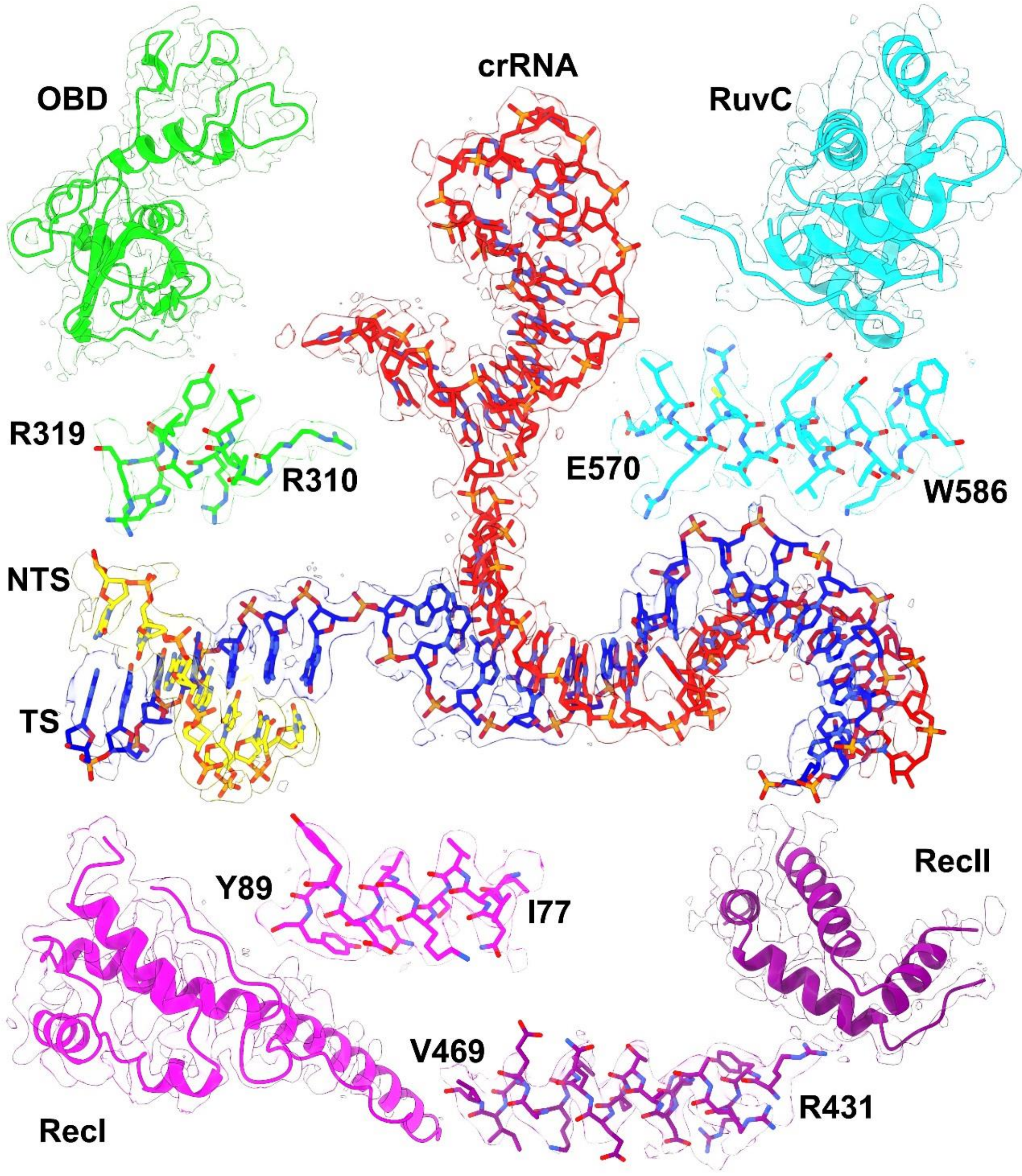

Fig. S3 CryoEM densities of each domain and the crRNA-dsDNA in the Cas12j-SF05^{crRNA}-dsDNA ternary complex.

Densities for indicated regions are shown in the context of the atomic model.

Supplementary Fig. S4

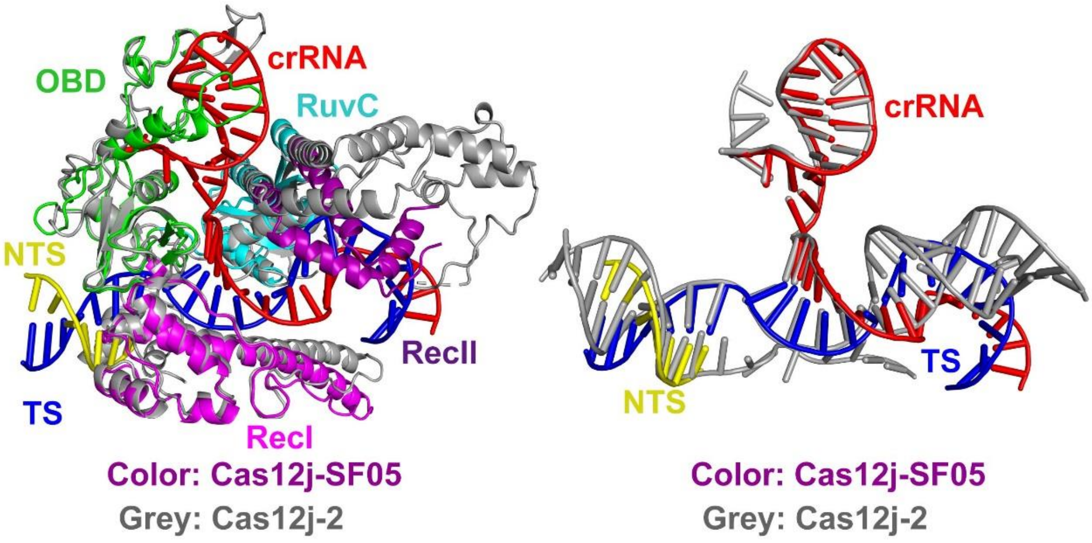

Fig. S4 Structure comparison of Cas12j-SF05 with Cas12j-2.

Structural comparison of the Cas12j-SF05^{crRNA}-dsDNA ternary complex (in color) and Cas12j-2^{crRNA}-dsDNA ternary complex (PDB 7LYS, in grey).

Supplementary Fig. S5

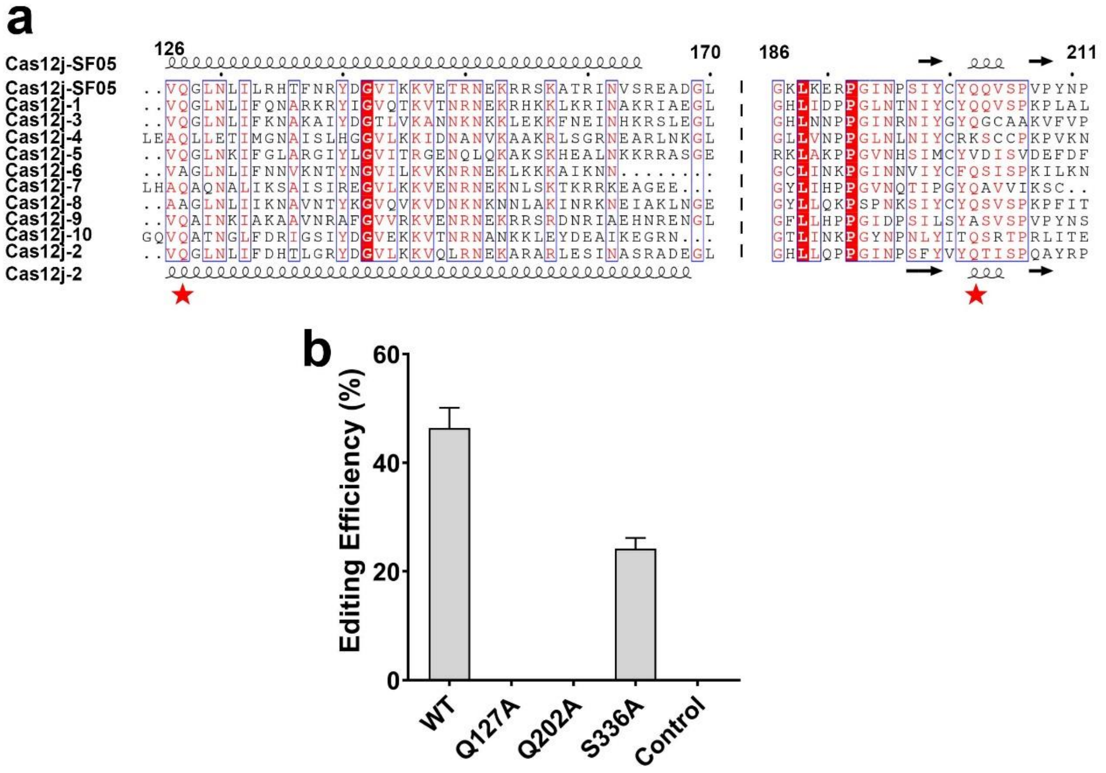

Fig. S5 Sequence alignment and mutagenesis analysis of residues involved in PAM recognition and target dsDNA unwinding by Cas12j-SF05 .

a. The highly conserved glutamines Q127 and Q202 responsible for PAM recognition are indicated by red stars.

b. Quantification of the activity of Cas12j-SF05 mutants based on *in vivo gene editing* assays (n = 3 independent reaction replicates; mean ± s.d.).

Supplementary Fig. S6

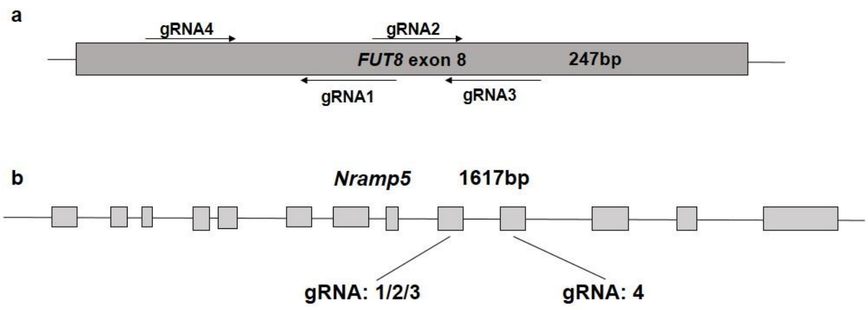

Fig. S6 *FUT8* gene (partial) and *Nramp5* gene (partial) map showing the locations of guide RNA target sites and the orientations of guide RNAs.

Supplementary Table S1

Table S1 Cryo-EM data collection, refinement and validation statistics.

	Cas12j-SF05^{crRNA}-dsDNA ternary complex
**Data collection and processing**	
Magnification	130,000
Voltage (keV)	300
Electron exposure (e$^{–}$/Å^{2})	50
Defocus range (μm)	1.5 to 2.5
Pixel size (Å)	0.66
Symmetry imposed	C1
Initial particle images (no.)	523,828
Final particle images (no.)	79,179
Map resolution (Å)	3.10
FSC threshold	0.143
**Refinement**	
Initial model used (PDB code)	*de-novo*
Model resolution (Å)	3.29
FSC threshold	0.5
Map sharpening *B* factor (Å^{2})	-83.8
Map Correlation Coefficient	0.83
Model composition	
Non-hydrogen atoms	5,321
Protein residues	498
Nucleotide base	65
B factor (Å^{2})	
Protein()	29.57
Nucleotide()	45.40
R.m.s. deviations	
Bond lengths (Å)	0.004
Bond angles (°)	0.857
Validation	
MolProbity score	1.83
Clash score	6.93
Poor rotamers (%)	0.24
Ramachandran plot	

Favored (%)	93.09
Allowed (%)	6.91
Disallowed (%)100%	0
PDB	8INB
EMDB	35595

Supplementary Data S1. Distribution of PAM sequence of Cas12j-SF05.

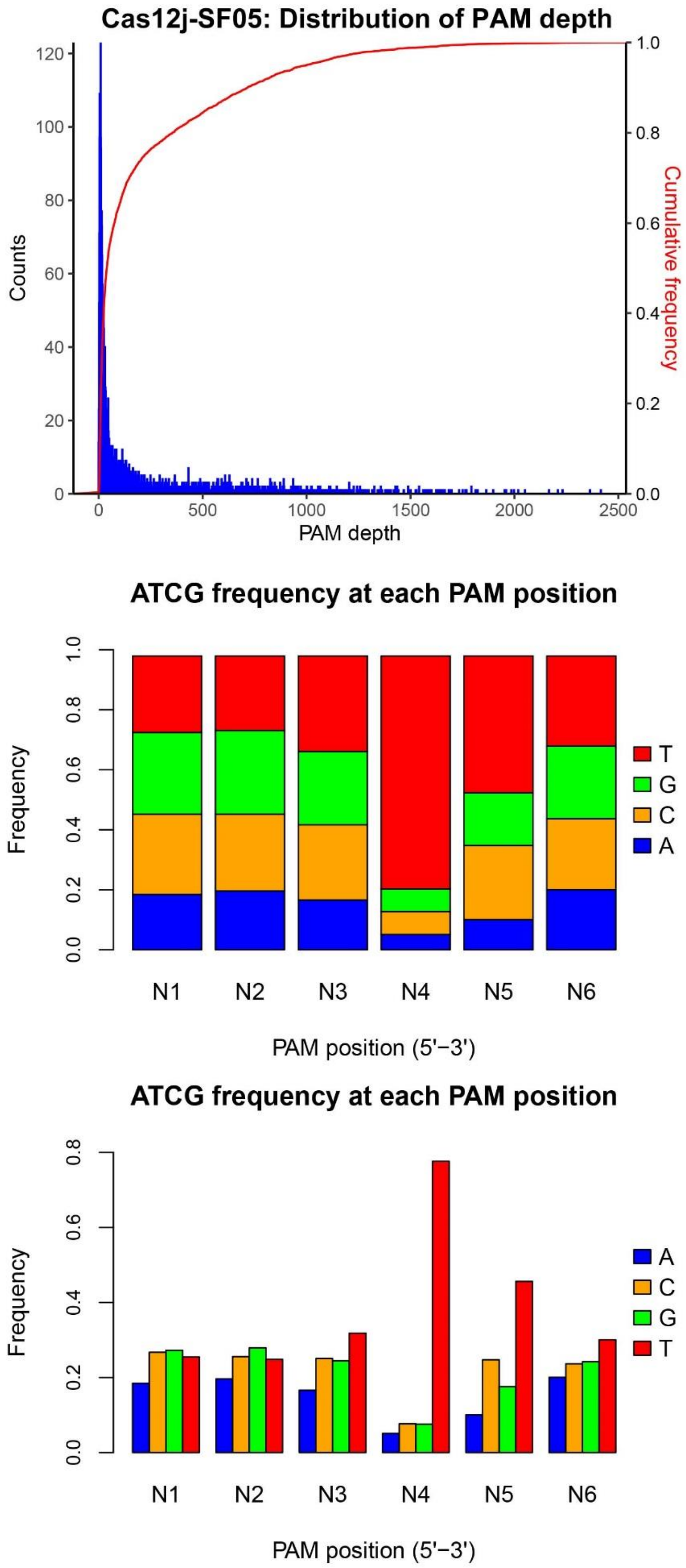

Supplementary Table S2. Oligonucleotides, target sequences, protein sequence, plasmids and protein sequences for phylogenic analysis used in this study.
